# Systematic review and meta-analysis of current guidelines, and their evidence base, on risk of renal function after administration of contrast medium for diabetic patients receiving metformin

**DOI:** 10.3389/fmed.2025.1547725

**Published:** 2025-06-26

**Authors:** Qinhui Xu, Weixing Huang, Qianyun Li, Tongan Bao, Hua Luo, Xiao Luo

**Affiliations:** ^1^Department of Radiology, Taizhou Hospital of Zhejiang Province Affiliated to Wenzhou Medical University, Taizhou, Zhejiang, China; ^2^General Surgical Department, Taizhou Hospital of Zhejiang Province, Zhejiang University, Taizhou, Zhejiang, China; ^3^Department of Nursing, Zhejiang University School of Medicine First Affiliated Hospital, Hangzhou, Zhejiang, China; ^4^Department of Orthopedics, Taizhou Hospital of Zhejiang Province Affiliated to Wenzhou Medical University, Taizhou, Zhejiang, China

**Keywords:** contrast agent, diabetic, renal function, CI-AKI, lactate

## Abstract

**Purpose:**

Our study aimed to determine through a meta-analysis whether continuing metformin use in diabetic patients receiving contrast agents would increase the risk of renal impairment and metabolic abnormalities.

**Methods:**

We searched the PubMed, EBSCO, Medline, and the Cochrane Central Register of Controlled Trials from the inception dates to March 2024. The included studies comparing metformin users and non-users during contrast agent administration in diabetic patients. Outcome measures included contrast-induced acute kidney injury (CI-AKI), serum creatinine, estimated glomerular filtration rate (eGFR), lactate level, and incidence of metabolic acidosis. We used odds ratio (OR) for dichotomous outcomes and weighted or standardized mean difference (WMD or SMD) for continuous outcomes, depending on scale consistency across studies.

**Results:**

Analysis involved 2 randomized controlled trials and 5 retrospective cohorts comprising 2020 patients. There were no significant differences between the metformin and non-metformin groups in CI-AKI incidence (OR: 0.87, 95% CI: 0.63–1.20), changes in renal function (serum creatinine: SMD: −0.15, 95% CI: −0.64–0.35; eGFR: WMD: 3.35, 95% CI: −1.60–8.29), incidence of metabolic acidosis (OR: 0.90, 95% CI: 0.57–1.43), and lactate levels (SMD: 0.29, 95% CI: −0.53–1.11). Sensitivity analysis excluding one study revealed a significant reduction in creatinine with metformin. Logistic regression meta-analysis showed that metformin use was not significantly associated with CI-AKI or metabolic acidosis, while contrast volume was the only consistent predictor of CI-AKI. Lower baseline CO_2_ was independently associated with increased risk of metabolic acidosis.

**Conclusions:**

Our analysis indicates that continuing metformin during contrast agent administration does not increase the risk of CI-AKI, acidosis, or eGFR compared to discontinuation or non-use of metformin. Additionally, continuation of metformin may be associated with a modest reduction in serum creatinine levels after contrast exposure. However, the limited quality of included studies may weaken the strength of these conclusions.

**Systematic review registration:**

https://www.crd.york.ac.uk/PROSPERO/view/CRD42023459602, identifier: CRD42023459602.

## Introduction

Type 2 diabetes mellitus (T2DM) represents one of the most significant health challenges of the twenty-first century, with a global prevalence on the rise. Data from the IDF Global Diabetes Map indicates that in 2025, the global adult diabetes population reached 589 million (11.1%) (1). T2DM can directly or indirectly lead to other more severe conditions, with ~30–40% of diabetic patients progressing to chronic kidney disease (2). Diabetic kidney disease stands as one of the leading causes of mortality among T2DM patients and is a primary contributor to end-stage renal disease worldwide, posing immense risks (3). Metformin serves as a frontline therapeutic agent for T2DM. In patients with preserved renal function, metformin does not exert direct nephrotoxic effects; however, in the presence of renal impairment, metformin can elevate blood lactate levels by inhibiting lactate metabolism, thereby resulting in metformin-associated lactic acidosis, with mortality rates reaching 30–50% (4). Additionally, patients seemingly with normal renal function but at risk of acute kidney injury (AKI), such as those with volume depletion, heart failure, sepsis, or exposure to nephrotoxic agents, also face an increased likelihood of developing metformin-associated lactic acidosis. Metformin increases lactate concentration primarily through its inhibitory effect on mitochondrial respiratory chain complex I, leading to reduced oxidative phosphorylation and a shift toward anaerobic glycolysis, thereby promoting lactate production (5). Additionally, metformin suppresses hepatic gluconeogenesis, particularly through inhibition of mitochondrial glycerophosphate dehydrogenase, leading to decreased hepatic lactate uptake and clearance (6). These effects are generally well-tolerated in patients with normal renal and hepatic function. However, in individuals with impaired renal function or tissue hypoxia, the reduced clearance and increased production of lactate can synergistically increase the risk of metformin-associated lactic acidosis (7). With the widespread use of contrast media (CM) in diagnostic and interventional procedures, contrast-induced acute kidney injury (CI-AKI) is becoming increasingly prevalent. While CI-AKI incidence ranges from 12 to 27% in the general population, the rate increases to 50% or more in patients with multiple risk factors (8). Consequently, early expert consensus and guidelines strongly recommend discontinuing metformin prior to CM use in T2DM patients (9, 10). However, emerging research suggests that the role of metformin in lactic acidosis may be overemphasized, with most complications related to T2DM serving as the primary culprits (11–13). In recent years, several guidelines regarding metformin have been revised. For instance, the American College of Radiology (ACR) revised its 2015 version recommendations for patients with an estimated glomerular filtration rate (eGFR) of 30–60 mL/min/1.73 m^2^. Previous versions advised discontinuation within 48 h post-surgery, while the new version (2023 edition) suggests continuation without cessation (9, 10, 14). While these updates are promising, they lack a strong evidence base, often relying on limited or outdated studies. This gap highlights the need for a comprehensive and up-to-date evaluation of the available evidence. Therefore, we conducted this study to rigorously assess whether discontinuing metformin prior to imaging in diabetic patients provides direct evidence on how to manage metformin use for T2DM patients undergoing CM administration. Our study aims to fill the gaps in the existing guidelines and provide more definitive evidence to guide clinical practice.

## Methods

The study conforms to the principles outlined in the Handbook of the Cochrane Collaboration (15), along with the guidelines established by the PRISMA statement (16). The protocol for this meta-analysis was registered on PROSPERO (Registration No: CRD 42023459602).

### Inclusion criteria

(1) Study compared patients with diabetes who were using metformin vs. those who were not using metformin during the administration of contrast agents;
(2) Study focused on relevant outcome;
(3) Diabetic patients (including T1DM & T2DM).

### Exclusion criteria

(1) Studies if they were letters, case reports, reviews, animal trials, or republished studies;
(2) Articles with missing data;
(3) Non-diabetic patient;
(4) Immunological diseases.

### Outcomes

The primary outcome was CI-AKI after the recent contrast medium injection. Secondary outcomes were serum creatinine, eGFR, lactate level, and incidence of metabolic acidosis after contrast medium used.

### Search strategy

Two of the authors performed the search in PubMed, EBSCO, Medline, and the Cochrane Central Register of Controlled Trials from the inception dates to March 2024, using the keywords “metformin” and “contrast medium” and “diabetes” and “(renal function OR serum creatinine OR eGFR OR lactate level OR metabolic acidosis)”.

### Data collection process

Two investigators used a standard data extraction form to extract all related data from selected studies independently. Data extracted included the first author's name, year of publication, country, type of study, sample size, age, and outcomes. Disagreements were resolved by consensus.

### Assessment of risk of bias and quality of evidence

Two researchers independently assessed the quality of all included studies based on Cochrane risk-of-bias criteria or the Newcastle–Ottawa scale (NOS) (17, 18).

### Data synthesis

The meta-analysis used Stata (version 17). Heterogeneity was assessed by the *Q*-test and *I*^2^-value. A random effects model was applied. Odds ratios (OR) with 95% CI were used for dichotomous outcomes. For continuous outcomes, weighted mean differences (WMDs) were used when measurement scales were consistent across studies, and standardized mean differences (SMDs) were applied when different scales were used. Statistical significance was set at *P* < 0.05.

## Results

**Figure 1:**
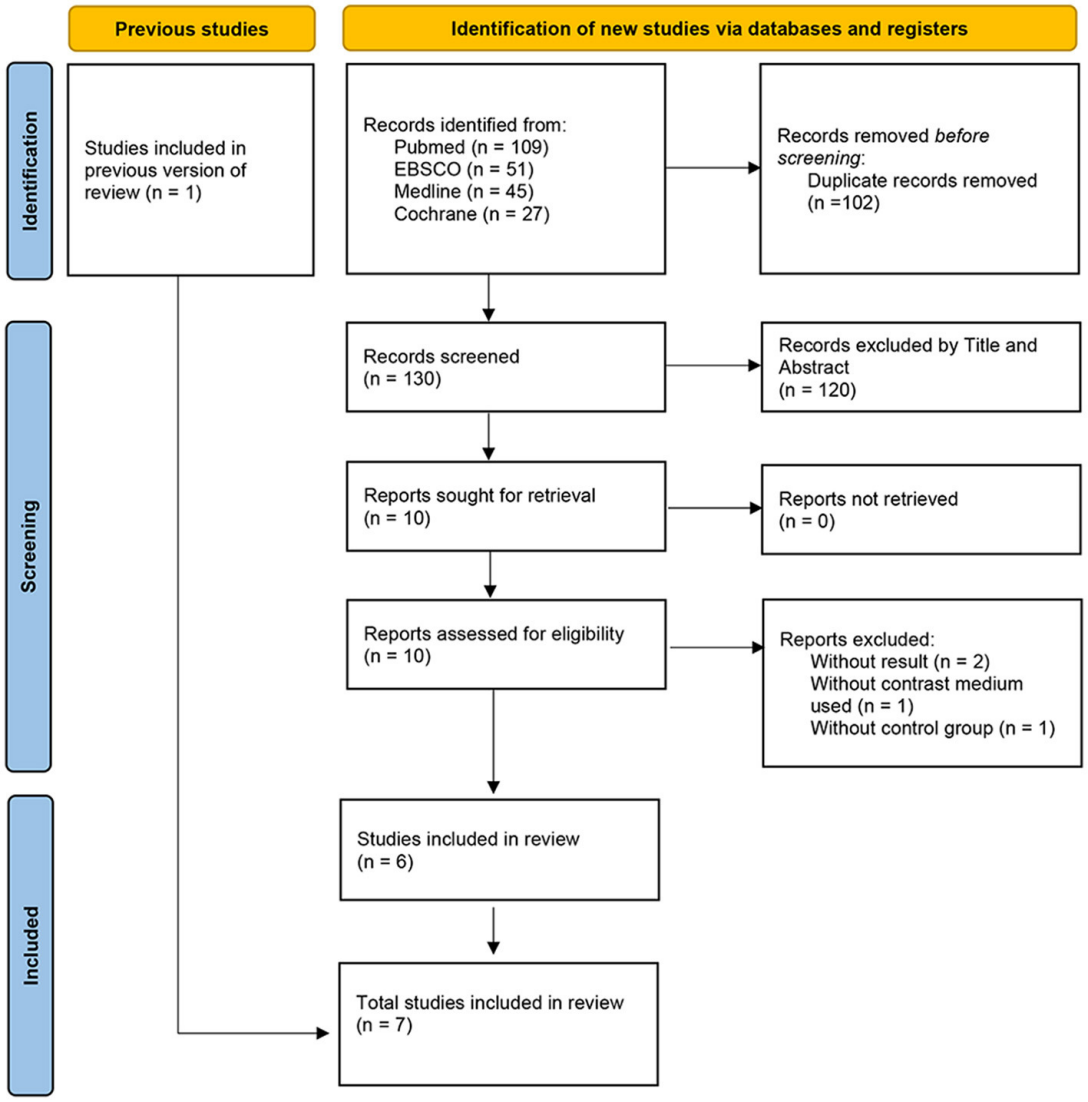
Flow diagram for search and selection of included studies.

A total of 232 potentially relevant articles were retrieved. After excluding 102 duplicate articles, a review of titles and abstracts of the remaining 130 articles led to the exclusion of 120 articles. Upon full-text reading of the remaining 10 articles, 4 articles were further excluded (2 clinical trials with no results, 1 article not using CM, and 1 article lacking a control group). Additionally, 1 previously conducted systematic review article was included, resulting in a total of 7 studies meeting our eligibility criteria (19–25). These studies comprised 2020 diabetic patients, among whom 893 continued metformin use during CM examinations, while 1,127 did not. The included 7 studies consisted of 2 RCTs and 5 retrospective cohort studies, with basic information about the included studies detailed in Table 1. Results indicated generally high quality across the included studies. The study flow diagram is depicted in Figure 1.

**Table 1:** Characteristics of included studies.

**Study**	**Design**	**Country**	**Participants**	**Duration**	**Treatment group**	**Control group**	**No. of subjects**		**Age**		**Outcomes**	**Risk of bias assessment tool (RCTs) or NOS (observational)**
							**Metformin**	**Non-metformin**	**Metformin**	**Non-metformin**		
Jung et al. (19)	RCS	Korea	T2DM	2015–2017	Metformin	Other oral hypoglycaemic agents	157	217	72.9 ± 9.1	72.5 ± 10.0	CI-AKI, LA, Scr, eGFR, metabolic acidosis	6
Kalkan et al. (20)	RCS	Turkey	Diabetic patients	2014–2019	Metformin	Non-metformin	148	195	61.3 ± 11.9	63.7 ± 12.5	CI-AKI	7
Kim et al. (21)	RCS	Korea	Diabetic patients	2012.01–2012.12	Metformin	Other oral hypoglycaemic agents	105	112	67.9 ± 10.6	65.3 ± 12.5	CI-AKI, Scr, metabolic acidosis	7
Namazi et al. (22)	RCT	Iran	Diabetic patients	2012.02–2012.11	Metformin	Non-metformin	83	79	61.5	60.1	LA, Scr, eGFR	High
Oktay et al. (23)	RCT	Turkey	T2DM	2016.01–2016.12	Metformin	Non-metformin	134	134	59.4 ± 7.7	61.4 ± 6.5	CI-AKI, LA, Scr, eGFR	High
Yu et al. (24)	RCS	China	T2DM	2008–2018	Metformin	Non-metformin	119	165	NA	NA	CI-AKI, Scr, eGFR	8
Zeller et al. (25)	RCS	France	T2DM	2001–2010	Metformin	Non-metformin	147	225	61 ± 11	65 ± 13	CI-AKI, Scr, eGFR	7

RCS, retrospective cohort study; RCT, Randomized Controlled Trial; NOS, Newcastle–Ottawa scale; LA, lactate; CI-AKI, contrast-induced acute kidney injury; eGFR, estimated glomerular filtration rate; Scr, serum creatinine; T2DM, Type 2 diabetes mellitus; NA, not applicable.

### CI-AKI

**Figure 2:**
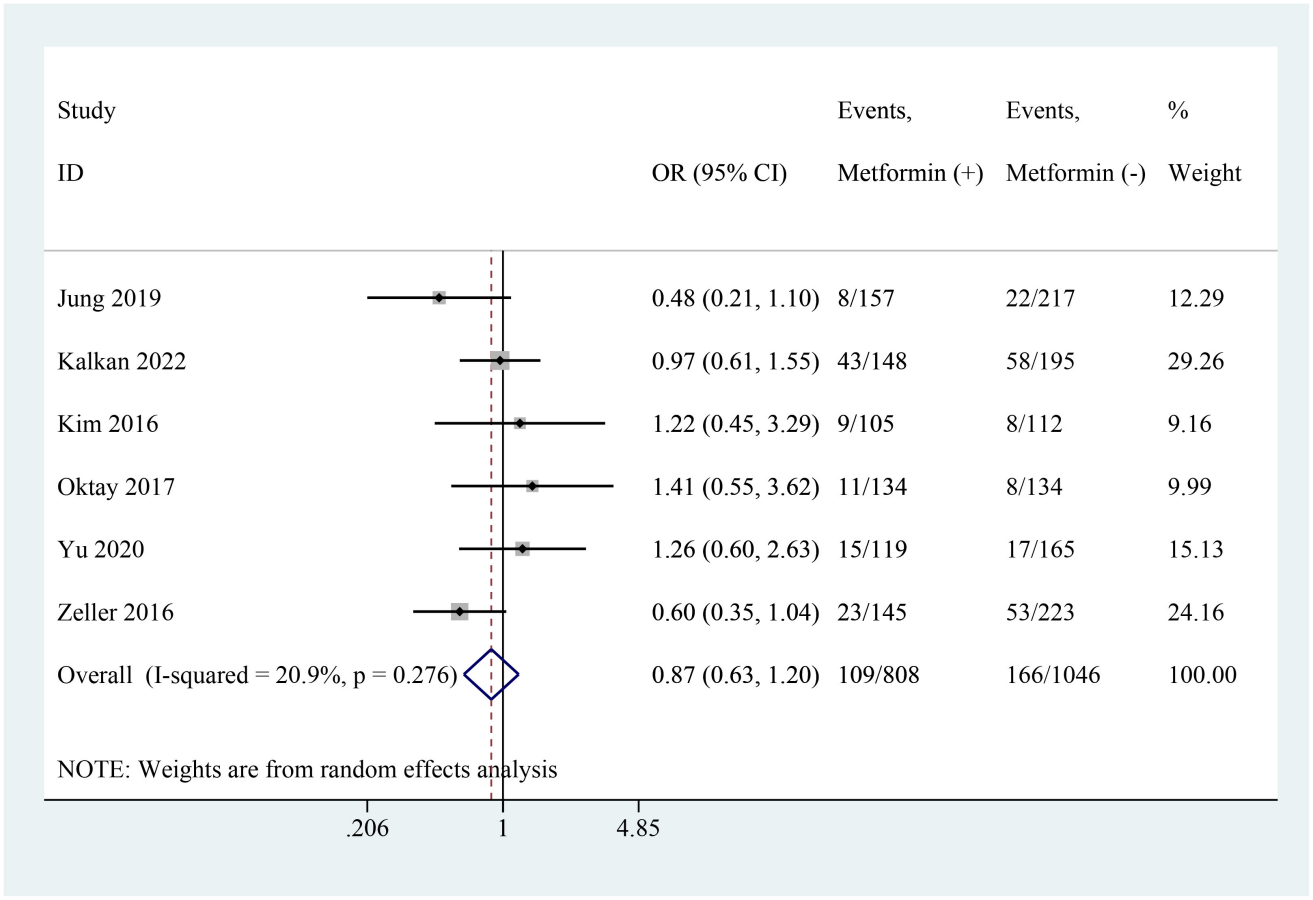
Pooled analysis of CI-AKI between the metformin and non-metformin.

In the 7 studies, 6 studies described the incidence rate of CI-AKI (19–21, 23–25). Their results consistently found that the risk of developing CI-AKI after CM administration was not associated with the use of metformin. The pooled analysis showed that there was no statistically significant increase in the risk of CI-AKI among patients continuing metformin use (OR: 0.87, 95% CI: 0.63–1.20, *I*^2^ = 20.9%, *P* = 0.404; Figure 2).

### Serum creatinine

**Figure 3:**
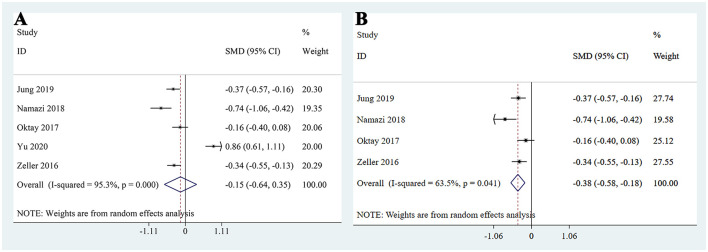
Pooled analysis of serum creatinine between the metformin and non-metformin **(A)** Overall pooled analysis; **(B)** Sensitivity analysis excluding the study by Yu et al. due to high heterogeneity.

A total of 5 studies reported serum creatinine levels (19, 22–25) among which 3 studies found that patients continuing metformin use showed decrease serum creatinine levels compared to the non-metformin group after receiving CM (19, 22, 25), while 1 studies found no difference between the two groups (23). In contrast, one study reported that continuing metformin was associated with increased serum creatinine levels (24). The pooled analysis indicated that there was no difference in creatinine levels between diabetic patients continuing metformin use and those not using metformin after CM (SMD: −0.15, 95% CI: −0.64–0.35, *I*^2^ = 95.3%, *P* = 0.562; Figure 3A). However, when the study by Yu et al. (24) was excluded in sensitivity analysis due to its methodological heterogeneity and large contribution to between-study variance, the results changed notably. The updated pooled analysis based on the remaining four studies showed a statistically significant reduction in serum creatinine levels in the metformin group (SMD: −0.38, 95% CI: −0.58 to −0.18; *I*^2^ = 63.5%, *P* < 0.001; Figure 3B).

### eGFR

**Figure 4:**
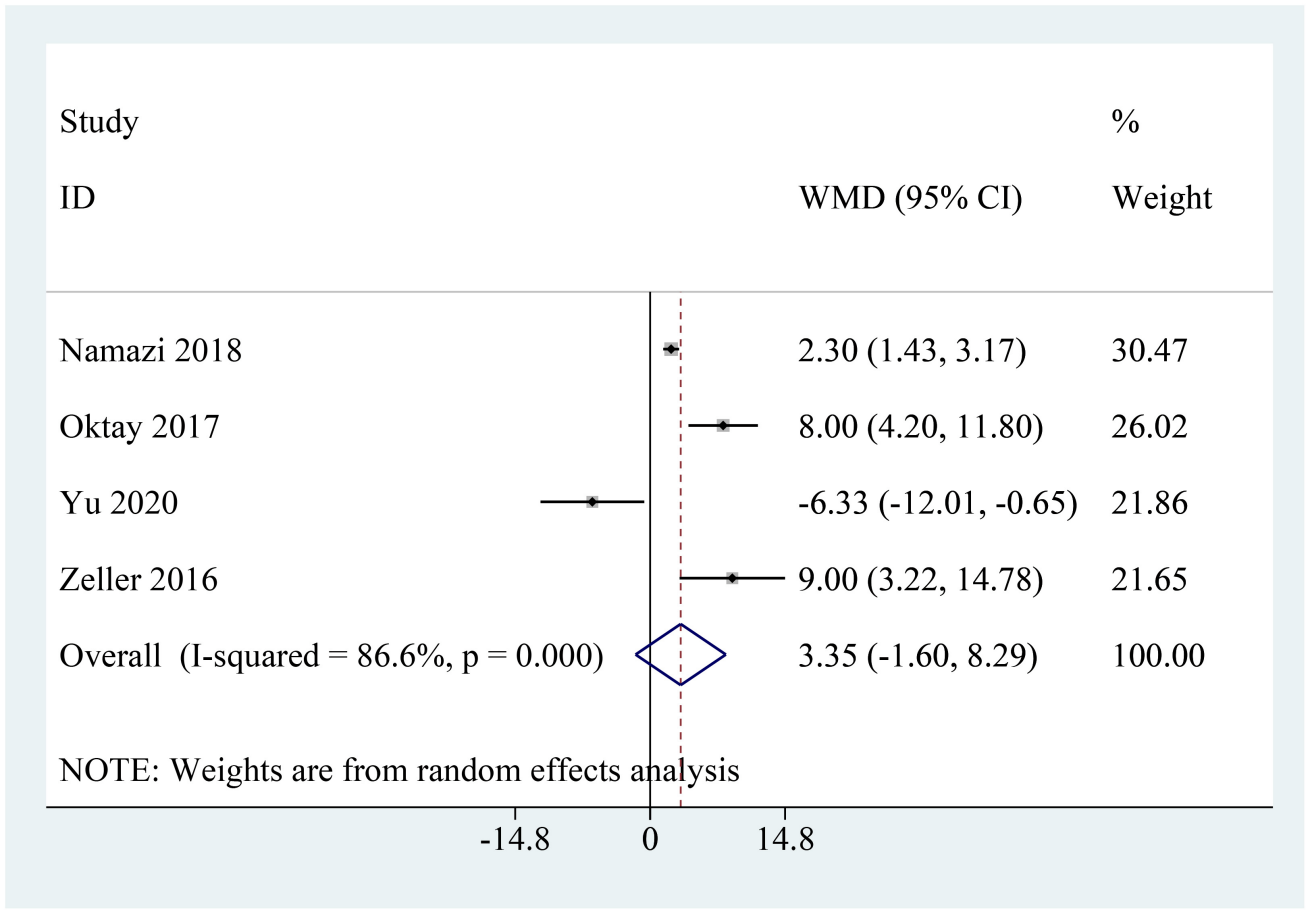
Pooled analysis of eGFR between the metformin and non-metformin.

Four studies reported on the eGFR situation (22–25). Among them, three studies found that the eGFR values in the metformin group were higher than those in the control group. However, Zeller et al.'s (25) study found that the eGFR values in the metformin group were lower than those in the group that discontinued metformin after receiving contrast agents. The pooled analysis showed that there was no statistically significant difference in eGFR change between patients continuing metformin use and those discontinuing metformin before and after contrast imaging (WMD: 3.35, 95% CI: −1.60–8.29, *I*^2^ = 86.6%, *P* = 0.184; Figure 4).

### Metabolic acidosis

**Figure 5:**
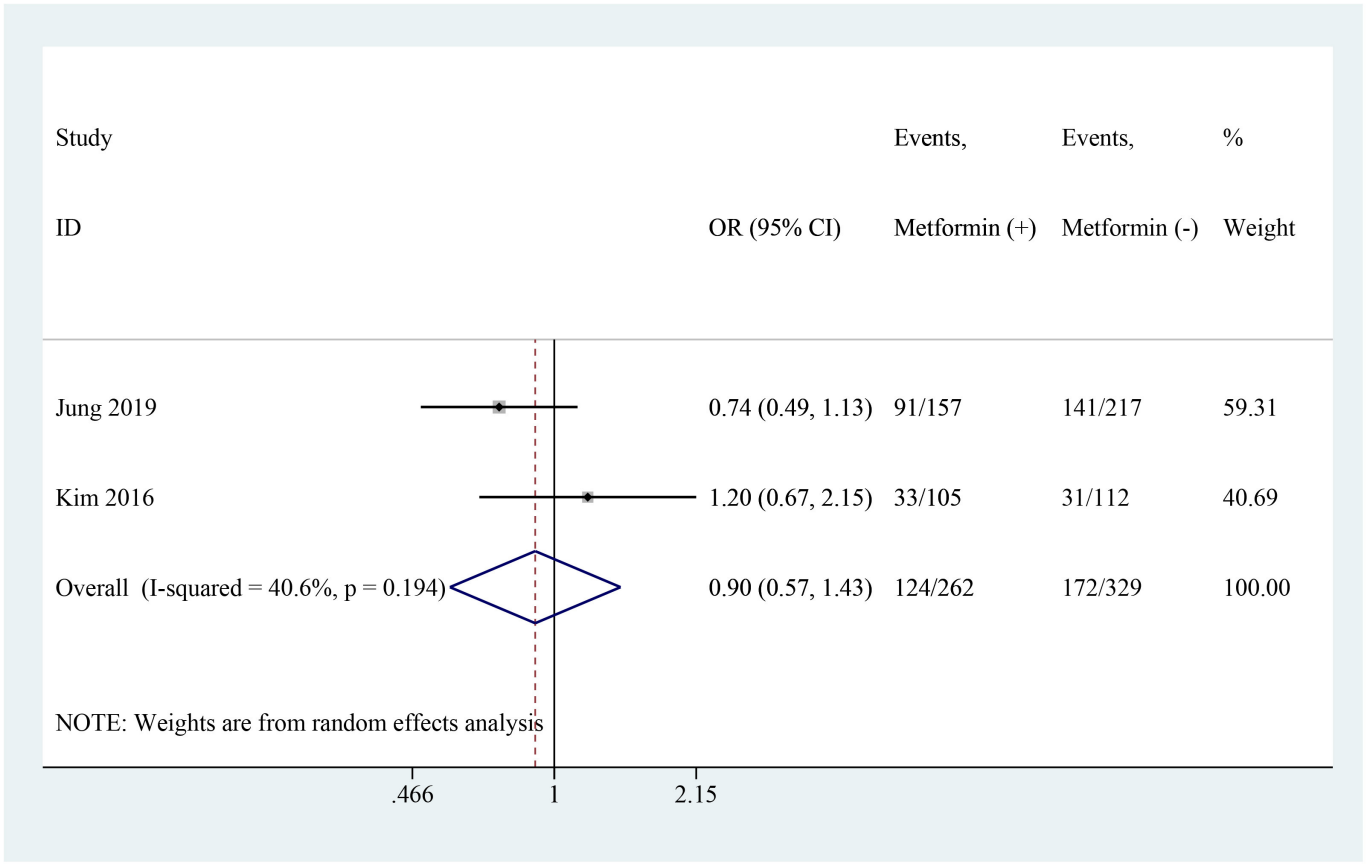
Pooled analysis of metabolic acidosis between the metformin and non-metformin.

Among the 7 studies, 2 studies reported the results of metabolic acidosis (19, 21). Both studies found no significant correlation between continuing metformin use during iodine contrast agent administration and metabolic acidosis. The pooled analysis indicated that there was no statistically significant increase in the risk of metabolic acidosis among patients continuing metformin use (OR: 0.90, 95% CI: 0.57–1.43, *I*^2^ = 40.6%, *P* = 0.661; Figure 5). To better understand patient-related risk factors for lactic acidosis, we further summarized baseline data from the included studies in Table 2. Key variables such as liver cirrhosis status, hemoglobin A1c (HbA1c), glucose, serum creatinine, eGFR, and CO_2_ levels were extracted when available.

**Table 2:** Baseline clinical characteristics and lactic acidosis–related risk factors of patients in included studies.

**Study**	**Liver cirrhosis**		**HbA1c (%)**		**Glucose (mg/dL)**		**Creatinine (mg/dL)**		**eGFR (mL/min)**		**CO**_**2**_ **(mmol/L)**	
	**Metformin**	**Non-metformin**	**Metformin**	**Non-metformin**	**Metformin**	**Non-metformin**	**Metformin**	**Non-metformin**	**Metformin**	**Non-metformin**	**Metformin**	**Non-metformin**
Jung et al. (19)	10.20%	10.60%	7.5 ± 1.7	7.4 ± 1.9	173 (129–247)^‡^	193 (135.6–269)^‡^	1.33 (1.18–1.48)^‡*^	1.43 (1.22–1.64)^‡^	48.6 (40.3–54.3)^‡*^	44.3 (37.8–50.8)^‡^	20.8 ± 4.2	20.6 ± 4.3
Kalkan et al. (20)	NS	NS	NS	NS	177 ± 97	170.3 ± 116	1.01 ± 0.74	1.03 ± 0.5	NS	NS	NS	NS
Kim et al. (21)	12.40%	10.70%	NS	NS	7.6 (5.7–11.7)^‡§^	7.8 (5.1–12.2)^‡§^	69.8 (54.8–87.5)^‡¶^	74.3 (60.1–95.5)^‡¶^	66.2 (50.9–87.9)^‡^	63.6 (47.8–92.4)^‡^	23.7 ± 4.1	23.7 ± 3.1
Namazi et al. (22)	NS	NS	NS	NS	NS	NS	NS	NS	NS	NS	NS	NS
Oktay et al. (23)	NS	NS	7 (5.6–13)^‡^	7.3 (5.5–12.9)^‡^	131 (87–271)^‡^	150 (90–328)^‡^	0.84 ± 0.18 mg/dL	0.84 ± 0.13	86 ± 18	81 ± 9	NS	NS
Yu et al. (24)	NS	NS	NS	NS	NS	NS	76 (66–86)^‡¶^	73 (61–84)^‡¶^	89 (73–104)^‡^	94 (72–113)^‡^	NS	NS
Zeller et al. (25)	NS	NS	7.5 (6.7–8.6)^‡^	7.4 (6.6–8.6)^‡^	13.7 (9.54–16.88)^‡§^	12.98 (9.18–16.58)^‡§^	87 (73–105)^‡*¶^	94 (77–120)^‡¶^	80 ± 26^*^	71 ± 29	NS	NS

HbA1c, Hemoglobin A1c; eGFR, estimated glomerular filtration rate; NS, not specified.

^*^Significant difference between the two groups.

^‡^Median (IQR).

^§^Unit: mmol/L.

^¶^Unit: μmol/L.

### Lactate level

**Figure 6:**
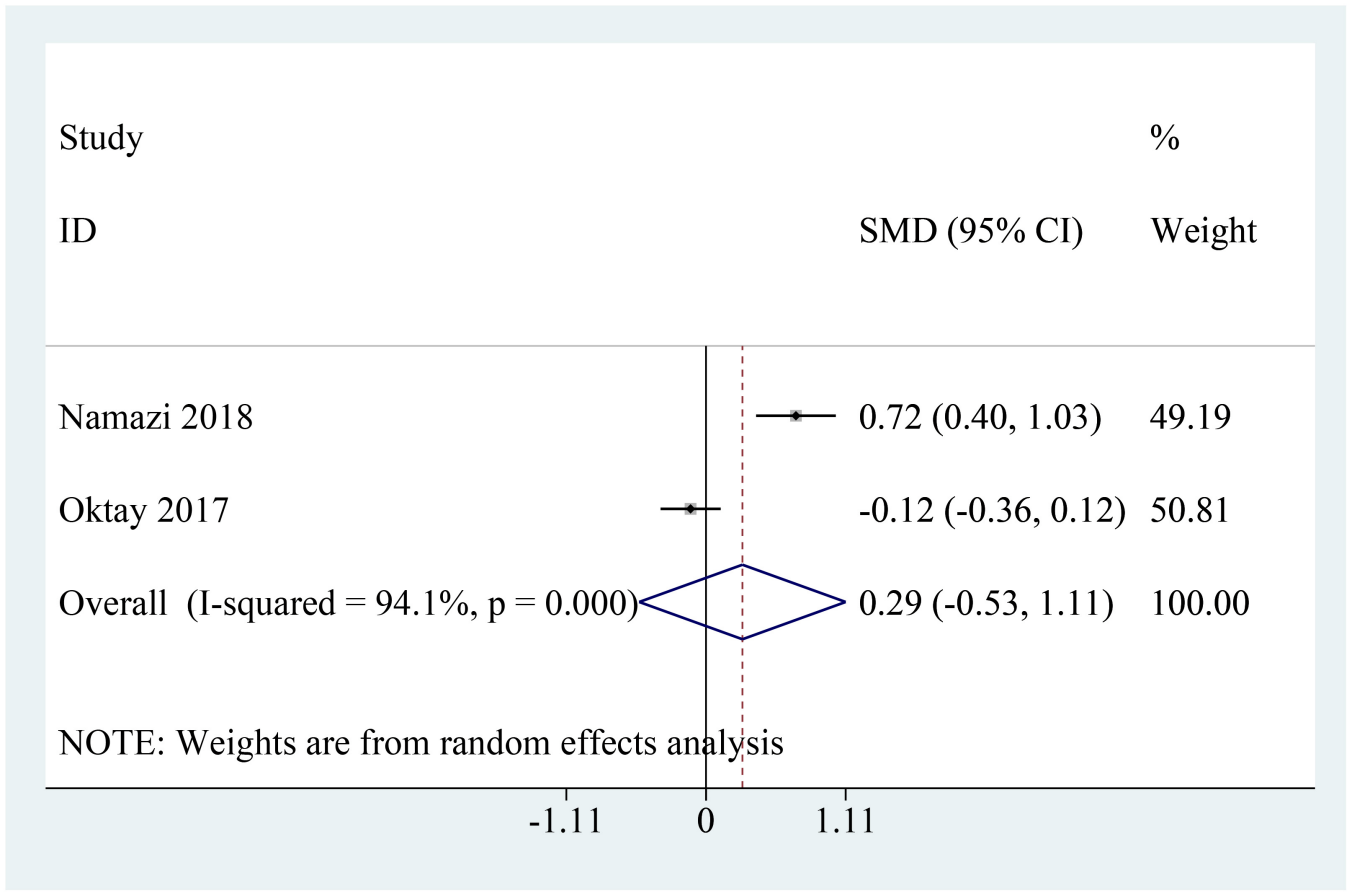
Pooled analysis of lactate level between the metformin and non-metformin.

Two studies reported on post-examination blood lactate levels (22, 23). Among them, Namazi et al. (22) found that continued use of metformin was associated with an increase in lactate levels, whereas Oktay et al. (23) reported no significant difference between the metformin and non-metformin groups. The pooled analysis indicated that there was no statistically significant difference in blood lactate levels after CM administration between patients continuing metformin use (SMD: 0.29, 95% CI: −0.53–1.11, *I*^2^ = 94.1%; *P* = 0.489, Figure 6).

### Meta-analysis of logistic regression of CI-AKI

**Figure 7:**
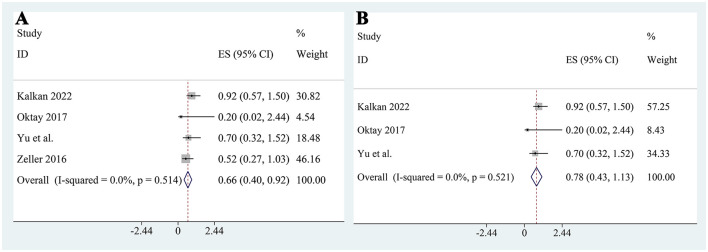
Pooled logistic regression analysis of the association between metformin use and CI-AKI. **(A)** Combined adjusted OR from all included studies; **(B)** Sensitivity analysis excluding Zeller et al.

**Figure 8:**
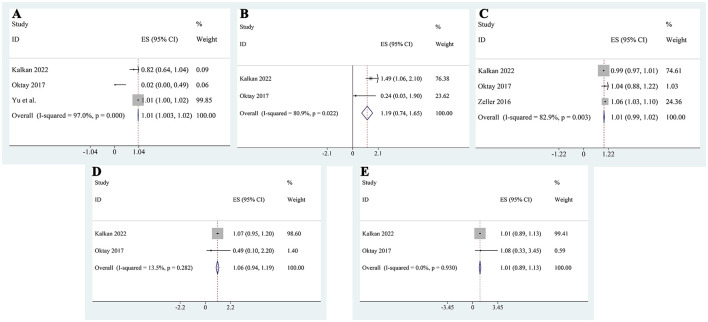
Pooled logistic regression results for additional covariates potentially associated with CI-AKI: **(A)** contrast volume; **(B)** baseline serum creatinine; **(C)** age; **(D)** glycated hemoglobin; **(E)** hemoglobin level.

Meta-analysis of logistic regression results from the included studies revealed that the use of metformin was not significantly associated with an increased risk of CI-AKI. The pooled adjusted OR was 0.66 (95% CI: 0.40–0.92; Figure 7A), suggesting a potential protective effect. However, sensitivity analysis showed that after excluding the study by Zeller et al. (25), the association was no longer statistically significant (pooled OR: 0.78, 95% CI: 0.43–1.13; Figure 7B). This result warrants careful interpretation. The study by Zeller et al. (25) did not adjust for baseline eGFR as a continuous variable in their regression model; instead, they used a binary variable (eGFR <30 mL/min/1.73 m^2^). Notably, the baseline eGFR was significantly higher in the metformin group compared to the control group. This imbalance might have artificially exaggerated the decline in renal function post-contrast in the metformin group, contributing to a biased protective effect. Therefore, the apparent significance of metformin in the initial pooled model is likely driven by this methodological limitation. In addition to metformin, we also analyzed other covariates that were included in at least two studies. Contrast volume was identified as a consistent and statistically significant independent risk factor for CI-AKI (pooled OR: 1.01, 95% CI: 1.003–1.02; Figure 8A), underscoring the importance of minimizing contrast exposure. Although baseline creatinine is a clinically important variable, only two studies included it as a covariate in their multivariable models, and the pooled analysis showed that its effect was not significant (pooled OR: 1.19, 95% CI: 0.74–1.65; Figure 8B), likely due to inconsistent adjustment methods and definitions. Other variables such as age (pooled OR: 1.01, 95% CI: 0.99–1.02; Figure 8C), glycated hemoglobin (pooled OR: 1.06, 95% CI: 0.94–1.19; Figure 8D), and hemoglobin level (pooled OR: 1.01, 95% CI: 0.89–1.13; Figure 8E) were also not significantly associated with CI-AKI. These findings suggest that, among the available covariates, contrast volume remains the most consistent predictor, while the role of other factors remains uncertain due to limited and heterogeneous reporting.

### Meta-analysis of logistic regression of metabolic acidosis

**Figure 9:**
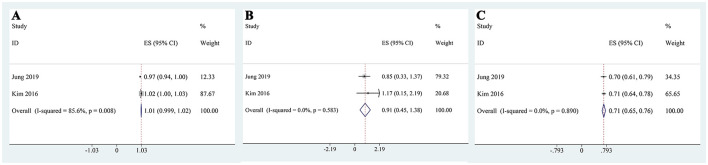
Pooled logistic regression results for predictors of metabolic acidosis: **(A)** age; **(B)** metformin use; **(C)** baseline CO_2_ level.

Our pooled analysis showed that neither age (Figure 9A) nor continuation of metformin (Figure 9B) use was significantly associated with the occurrence of metabolic acidosis. In contrast, baseline CO_2_ levels demonstrated a significant inverse association with metabolic acidosis risk. Specifically, the pooled logistic regression analysis indicated that lower CO_2_ levels were independently associated with a higher risk of metabolic acidosis (OR: 0.71, 95% CI: 0.65–0.76; Figure 9C), highlighting CO_2_ as a potential protective factor.

### Publication bias

With fewer than 10 trials, publication bias was not assessed using funnel plots.

## Discussion

The study found no significant differences in the occurrence of CI-AKI, lactate levels, incidence of acidosis, and alterations in renal function parameters between diabetic patients who are using metformin and those who are not. However, sensitivity analysis showed that after excluding the study by Yu et al., which introduced methodological heterogeneity, the pooled results indicated a statistically significant reduction in serum creatinine levels among patients who continued metformin. In addition, meta-analysis of logistic regression results revealed that metformin use was not independently associated with an increased risk of CI-AKI. The pooled adjusted OR suggested a potential protective effect, though this association became no significant after excluding the study by Zeller et al. (25), which did not adjust for baseline eGFR as a continuous variable. Among other covariates, contrast volume consistently emerged as a statistically significant independent risk factor for CI-AKI, while age, baseline creatinine, HbA1c, and hemoglobin were not associated. For metabolic acidosis, logistic regression meta-analysis demonstrated that neither age nor metformin continuation was associated with increased risk. In contrast, lower baseline CO_2_ levels were significantly associated with a higher risk of metabolic acidosis, highlighting CO_2_ as a potential independent predictor. Previous meta-analyses have also examined the relationship between metformin use and the occurrence of CI-AKI in patients undergoing iodine contrast agent examinations (26–28). Their conclusions indicated that continued use of metformin was not associated with CI-AKI. However, these studies primarily focused on patients taking metformin, whereas our meta-analysis specifically compared diabetic patients taking metformin with those who were not. Metformin is best known for its role in lowering blood glucose levels in diabetic patients (29). As research advances, investigators have discovered significant therapeutic effects of metformin in reducing cardiovascular events and combating obesity, among other benefits (30–32). Furthermore, there is increasing evidence suggesting potential benefits of metformin in conditions such as cancer, neurodegenerative diseases, metabolic syndrome, polycystic ovary syndrome, aging, and COVID-19 (33–39).

Diabetic patients have a higher susceptibility to developing lactic acidosis and renal function impairment compared to non-diabetic individuals (40). Poor glycemic control and abnormal energy metabolism contribute to increased lactate production, while impaired kidney function reduces lactate clearance. Additionally, diabetic patients may experience both ketoacidosis and lactic acidosis simultaneously, leading to more severe accumulation of acidic substances in the body (41). However, the population solely taking metformin includes non-diabetic individuals as well. Therefore, when Qiao et al. combined data from diabetic and non-diabetic patients for analysis, the study exhibited considerable heterogeneity, which undermined the credibility of its conclusions (28).

The belief that metformin should be discontinued in patients undergoing contrast agent treatment arises from the association of metformin with lactic acidosis and CI-AKI. This perspective stems from phenformin, a drug related to metformin, which was found to increase hepatic lactate production, leading to lactic acidosis and subsequently withdrawn from the market starting in 1978. Despite the similarity in their names, the chemical structure of metformin is significantly different. Metformin can inhibit hepatic gluconeogenesis without altering lactate turnover (42). A community-based cohort study involving around 1 million diabetic patients in the United States also indicates that metformin use is associated with acidosis only when the eGFR is below 30 mL/min/1.73 m^2^ (27, 43). Furthermore, emerging evidence indicates that in diabetic patients receiving metformin treatment, the majority of cases of lactic acidosis cannot be attributed to metformin toxicity (12, 13). Lactic acidosis can occur in non-diabetic patients in various conditions such as sepsis, hepatic failure, and renal failure. In fact, almost all reported cases of metformin-associated lactic acidosis occur in patients with comorbidities. Our study's results also confirm that metformin is not associated with the occurrence of lactic acidosis. The Korean Diabetes Association have reached a consensus on the use of metformin in type 2 diabetes complicated by renal insufficiency, particularly when these patients undergo imaging studies with CM. Renal function should be assessed before any CM-related procedure (44). Metformin is safe with eGFR ≥45; use ≤ 1,000 mg daily if eGFR is 30–44. It is contraindicated if eGFR <30 mL/min/1.73 m^2^. As the included studies did not specifically specify populations based on glomerular filtration rate, with Oktay et al. and Zeller et al. analyzing populations with eGFR <60 mL/min/1.73 m^2^ (23, 25), there were no reports on populations with glomerular filtration rates <30 mL/min/1.73 m^2^, so subgroup analysis based on different eGFR levels could not be performed.

## Limitations

This study has several limitations. First, apart from the 2 RCT studies, the remaining 5 studies were retrospective cohort studies. While their NOS scores were all >6 points, retrospective cohort studies are subject to risks of information bias and recall bias compared to RCT. Due to the inability to control confounding factors, their internal validity is lower, which may lead to biased results. Second, the included populations exhibited significant heterogeneity. In studies by Kalkan et al. (20) and Kim et al. (21), the creatinine levels in the non-metformin group were higher than those in the metformin group, reducing the accuracy and credibility of the combined effects. Third, since most diabetic patients often have multiple comorbidities, including hypertension, and may require antihypertensive medications, these drugs may also influence the study results (20). Forth, different trails may have treated the control group differently. Some studies suspended metformin intake before imaging, while in other studies, the control group took other antidiabetic medications. Fifth, subgroup analyses based on baseline eGFR and metformin dosage were not feasible due to inconsistent and incomplete reporting across studies. We acknowledge the value of such stratification and recommend future studies provide standardized data to enhance clinical applicability.

## Conclusion

No evidence suggests that continuing metformin during contrast medium administration increases the risk of CI-AKI, lactic acidosis, or worsening eGFR compared to those who discontinue or do not use metformin. In contrast, continuation of metformin may be associated with a modest reduction in serum creatinine following contrast exposure. For patients with eGFR <30 mL/min/1.73 m^2^, safety data is insufficient, requiring further research. More large-scale RCTs are needed to confirm these findings.

## Data Availability

The raw data supporting the conclusions of this article will be made available by the authors, without undue reservation.
